# Early Detection of *Botrytis cinerea* Infection in Cut Roses Using Thermal Imaging

**DOI:** 10.3390/plants12244087

**Published:** 2023-12-06

**Authors:** Suong Tuyet Thi Ha, Yong-Tae Kim, Byung-Chun In

**Affiliations:** Department of Smart Horticultural Science, Andong National University, Andong 36729, Republic of Korea; tuyetsuongha@gmail.com (S.T.T.H.); qkfkadpc@anu.ac.kr (Y.-T.K.)

**Keywords:** gray mold disease, petal temperature, postharvest, rose, thermography, vase life

## Abstract

*Botrytis cinerea* (*B. cinerea*) causes gray mold disease (GMD), which results in physiological disorders in plants that decrease the longevity and economic value of horticultural crops. To prevent the spread of GMD during distribution, a rapid, early detection technique is necessary. Thermal imaging has been used for GMD detection in various plants, including potted roses; however, its application to cut roses, which have a high global demand, has not been established. In this study, we investigated the utility of thermal imaging for the early detection of *B. cinerea* infection in cut roses by monitoring changes in petal temperature after fungal inoculation. We examined the effects of GMD on the postharvest quality and petal temperature of cut roses treated with different concentrations of fungal conidial suspensions and chemicals. *B. cinerea* infection decreased the flower opening, disrupted the water balance, and decreased the vase life of cut roses. Additionally, the average temperature of rose petals was higher for infected flowers than for non-inoculated flowers. One day before the appearance of necrotic symptoms (day 1 of the vase period), the petal temperature in infected flowers was significantly higher, by 1.1 °C, than that of non-inoculated flowers. The GMD-induced increase in petal temperature was associated with the mRNA levels of genes related to ethylene, reactive oxygen species, and water transport. Furthermore, the increase in temperature caused by GMD was strongly correlated with symptom severity and fungal biomass. A multiple regression analysis revealed that the disease incidence in the petals was positively related to the petal temperature one day before the appearance of necrotic symptoms. These results show that thermography is an effective technique for evaluating changes in petal temperature and a possible method for early GMD detection in the cut flower industry.

## 1. Introduction

The fungus *Botrytis cinerea* is a necrotrophic pathogen that causes gray mold disease (GMD) in various vegetable, fruit, and ornamental crops during both cultivation and postharvest handling [1,2]. Because cut flowers are maintained in a low temperature and high humidity environment over approximately 3 to 7 days when exported, huge economic damage may occur due to disposal or loss of marketability due to GMD appearance upon arrival [3]. Among ornamental crops, cut roses (*Rosa hybrida* L.) are susceptible to GMD, and their vase life (VL) is often shortened by this disease [4,5,6,7]. The symptoms of GMD in cut rose flowers include discoloration, spotting, and wilting [1,8]. Infection with *B. cinerea* often triggers defensive responses, including the production of reactive oxygen species (ROS) and phytohormones such as jasmonic acid, salicylic acid, and ethylene [9,10,11]. In cut roses, *B. cinerea* infection activated ethylene biosynthesis and signaling pathways, leading to the activation of ethylene response factors in rose petals [6]. Ethylene induced the development of *B. cinerea* infection in the cut rose flowers directly binding to the receptors or indirectly by promoting the ethylene response in the host tissues [6]. *B. cinerea* can also block the vascular tissues of plants, impairing the transport of nutrients and water to flowers. This can result in nutrient deficiency and early wilting symptoms in infected plants [12,13]. Dry transport has been implemented as an alternative to wet transport for cut rose flowers because of a reduction in space (thus cheaper), in flower bud opening (thus maturity stage is little affected), and a decrease in *B. cinerea* growth [3,14]. Although this approach effectively curbs the growth of *B. cinerea* in cut roses during transport, it also leads to water stress, reducing VL and flower freshness. Therefore, a screening system that can rapidly predict and determine the occurrence of GMD at an early stage in cut flowers is required. Currently, the selection and grading of cut roses primarily rely on manual labor, making it exceedingly challenging to detect GMD in its early stages. Furthermore, the lack of visible symptoms during the initial phase of infection in rose petals (penetration stages) complicates visual detection of the disease in greenhouses and during postharvest storage and transport [1,8].

Thermal imaging is an analytical technique in which temperature variation is visualized by colors representing intensity, using measurements of infrared wavelengths emitted from the surface of the subject rather than displaying the actual object [15,16]. Initially developed for industrial purposes, thermal imaging cameras are widely applied in the fields of electricity, process control, medicine, and agriculture [17,18,19]. In postharvest management, thermography has been utilized to assess quality and detect disease infections by monitoring temperature changes in horticultural products through thermal imagery [18,20,21,22,23,24]. This non-destructive approach for quality evaluation involves establishing a relationship between temperature characteristics and quality changes in fresh agricultural products, allowing for the identification of the physiological state of plants. Researchers have also explored the application of thermography to detect pre-symptomatic signs of diseases in various vegetables and fruits such as cucumbers, apples, pears, and grapevines [25,26,27,28,29,30].

Thermography has previously been used to detect powdery mildew and GMD in potted rose plants [23,24]. These studies have shown that thermal imaging enables the early detection of diseases in potted rose plants based on observations of temperature changes in leaves and flower petals following fungal infection [23,24]. However, the use of thermal imaging for the early detection of *B. cinerea* infection in cut roses has not been investigated. Therefore, the aim of this study was to explore the effectiveness of thermal imaging for the estimation of petal temperature changes in cut roses during GMD infection, providing a basis for the detection and prediction of GMD in cut roses based on thermal imaging. The postharvest quality, longevity, and petal temperature of the cut flowers were monitored during the transport and vase periods after *B. cinerea* inoculation. Furthermore, we examined the expression levels of genes related to ethylene biosynthesis, ROS, and water transport in petals to assess whether changes in petal temperature due to *B. cinerea* infection are related to plant defense mechanisms and tissue water content.

## 2. Results

### 2.1. Effects of B. cinerea Infection on the Postharvest Quality, Disease Index, and Petal Temperature of Cut Roses

**Figure 1:**
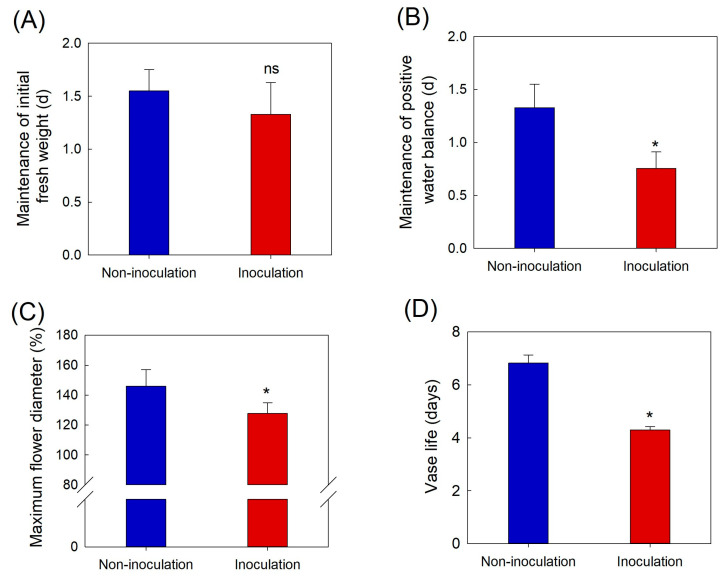
Effects of *Botrytis cinerea* inoculation on initial fresh weight (**A**), positive water balance (**B**), flower opening (**C**), and vase life (**D**) of cut rose flowers. Cut flowers were sprayed with 30 mL of *Botrytis cinerea* suspension (10^5^ conidia mL L^−1^) to induce GMD. Non-inoculated flowers were sprayed with 30 mL of distilled water. Data are shown as means ± SE (*n* = 24). ns and asterisks (*) represent non-significant and significant differences between non-inoculation and inoculation, as determined by Student’s *t*-test at *p* < 0.05.

Changes in water relations, flower opening, vase life, disease index (DI), and petal temperature were analyzed daily to assess the influence of *B. cinerea* infection on the postharvest quality. GMD infection did not affect the maintenance of the initial fresh weight of cut roses (Figure 1A). However, the development of GMD in the petals disrupted the water balance and decreased flower opening and VL of cut roses (Figure 1B–D).

**Figure 2:**
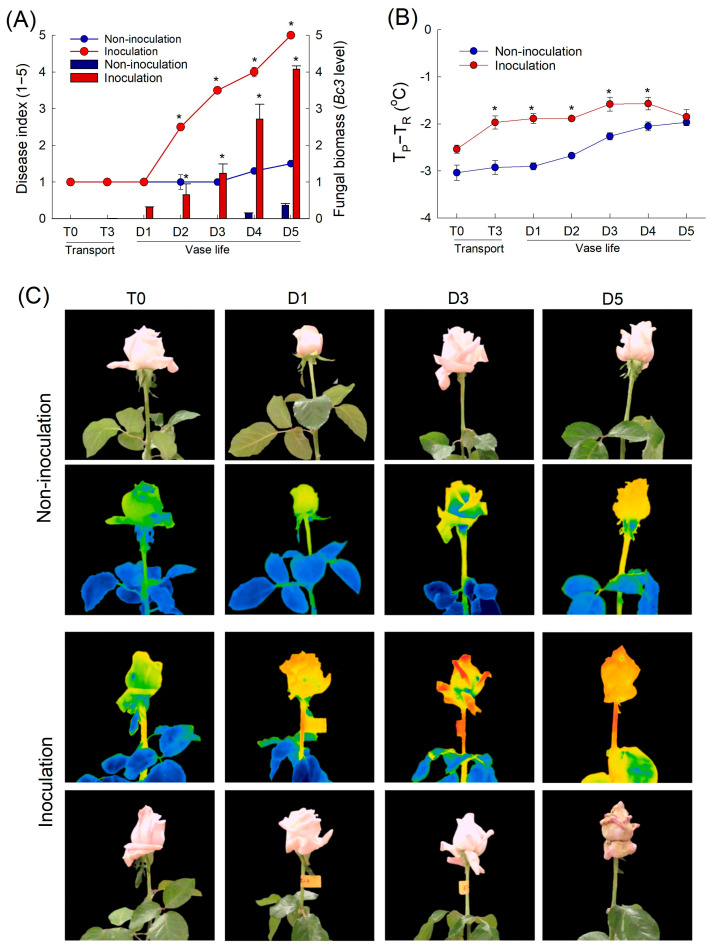
Progression of gray mold disease in cut roses assessed as the disease index and fungal biomass (evaluated by *Bc3* level) following the inoculation of *Botrytis cinerea* (**A**), effects of *Botrytis cinerea* infection on petal temperature difference (T_P_-T_R_) of cut roses (**B**), and thermal images of non-inoculated and inoculated cut roses (**C**). T_P_ and T_R_ are the temperature of petals and reference plate. Cut flowers were sprayed with 30 mL of *Botrytis cinerea* suspension (10^5^ conidia mL^−1^) to induce gray mold disease. Non-inoculated flowers were sprayed with 30 mL of distilled water. Disease index was classified into five levels as 1, none; 2, slight symptoms (≤3%); 3, moderate symptoms (3–10%); 4, severe symptoms (11–50%); and 5, death of flowers (>50%). Disease index, *Bc3* level, and petal temperature were evaluated on days 0 and 3 of transport (T0 and T3) and days 1–5 (D1–D5) of vase period. Data are shown as means ± SE (*n* = 24 for disease index, 12 for petal temperature and *Bc3* level). Asterisk (*) represent difference between non-inoculation and inoculation, as determined by Student’s *t*-test at *p* < 0.05.

Small necrotic lesions were visually detected in infected flowers on day 2 of the vase period. These small lesions developed into larger brown spots on the infected petals, eventually causing the infected petals to turn brown. The severity of petal symptoms increased over time; by day 5 of the vase period, all petals were macerated by GMD (Figure 2A). The increase in disease index (DI) was related to the fungal biomass (evaluated by *Bc3* levels) detected in the rose petals (Figure 2A). No disease symptoms were observed in non-inoculated cut flowers until day 3 of the vase period. Slight disease symptoms appeared in non-inoculated flowers on days 4 and 5 after cut roses displayed wilted petals (Figure 2A).

*B. cinerea* inoculation resulted in an increase in the average temperature of rose petals compared with that of non-inoculated flowers (Figure 2B,C). One day before the appearance of the first visual disease symptoms (day 1 of the vase period), the average petal temperature of infected flowers was 1.1 °C higher than that of non-inoculated flowers (Figure 2B). The petal temperature of infected flowers increased until day 4 and then tended to decrease at the late stage of disease infection (day 5) (Figure 2B). The petal temperature of non-inoculated flowers showed a slight increase on days 4 and 5 of the vase period (Figure 2B,C). These results suggested that thermal imaging can be used to effectively distinguish between healthy flowers and *B. cinerea*-infected flowers.

### 2.2. Associated between the GMD-Induced Temperature Increase and Plant Defense and Water Relation

**Figure 3:**
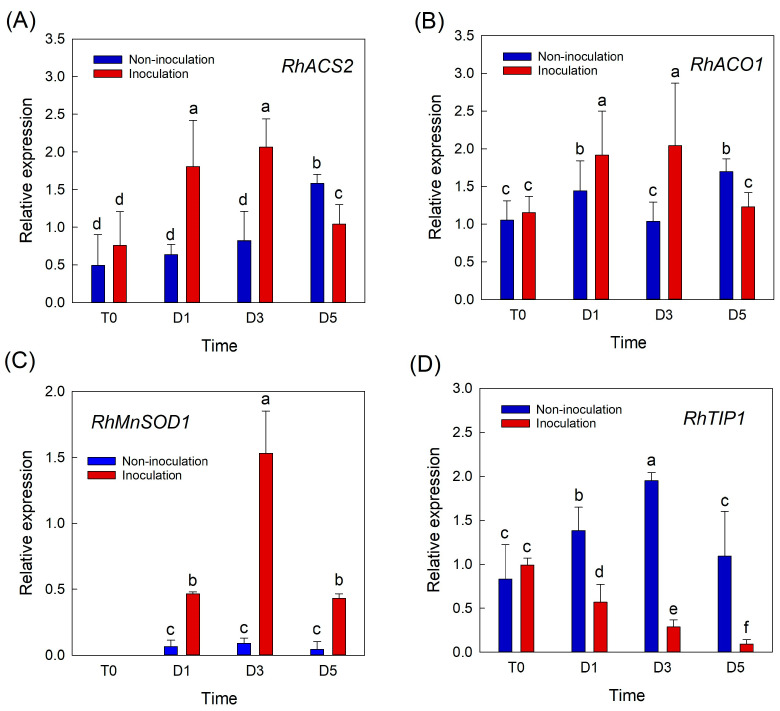
Effects of *Botrytis cinerea* infection on changes in relative expression of genes related to ethylene, ROS, and water transport in cut roses. *RhACS2* and *RhACO1*, ethylene biosynthesis genes (**A**,**B**); *RhMnSOD1*, ROS-related gene (**C**); and *RhTIP1*, aquaporin-related gene (**D**). Gene expression levels in cut roses were analyzed at day 0 of transport (T0) and at days 1 (D1), 3 (D3), and 5 (D5) of the vase period. Cut flowers were sprayed with 30 mL of *B. cinerea* suspension (10^5^ conidia mL^−1^) to induce GMD. Non-inoculated flowers were sprayed with 30 mL of distilled water. Data are shown as means ± SE (*n* = 6). Different letters above bars indicate statistically significant differences among treatments at *p* = 0.05 based on Duncan’s multiple range test.

To further evaluate whether the increase in petal temperature after *B. cinerea* inoculation was related to the plant defense system and water relation of cut roses, the expression patterns of genes related to ethylene biosynthesis (*RhACS2* and *RhACO1*), ROS (*RhCu/ZnSOD1* and *RhMnSOD1*), and water transport (*RhTIP1*) were monitored in rose petals. The selection of these genes for the experiments was based on previous studies [6,11,12]. The expression levels of the two ethylene biosynthesis-related genes were higher in inoculated flowers than in non-inoculated flowers on days 1 and 3 of the vase period (Figure 3A,B). On day 5, the mRNA levels of ethylene biosynthesis-related genes in infected flowers were lower than those in inoculated flowers, possibly because the necrotic tissues lost the ability to synthesize ethylene (Figure 3A,B). *B. cinerea* infection also increased the mRNA level of *RhMnSOD1* in rose petals during the vase period (Figure 3C). The accumulation of *RhCu/ZnSOD1* in petals was low but was higher in inoculated flowers than in non-inoculated flowers (Supplementary Figure S1). Unlike the ethylene biosynthesis- and ROS-related genes, the expression of *RhTIP1* in rose petals decreased rapidly in *B. cinerea*-infected flowers (Figure 3D). These data indicate that the increase in petal temperature by *B. cinerea* infection was closely associated with decreased water content in petals and the activation of the plant’s defense response.

### 2.3. Effect of Inoculum Concentrations of B. cinerea and Chemical Treatments on Petal Temperature

**Figure 4:**
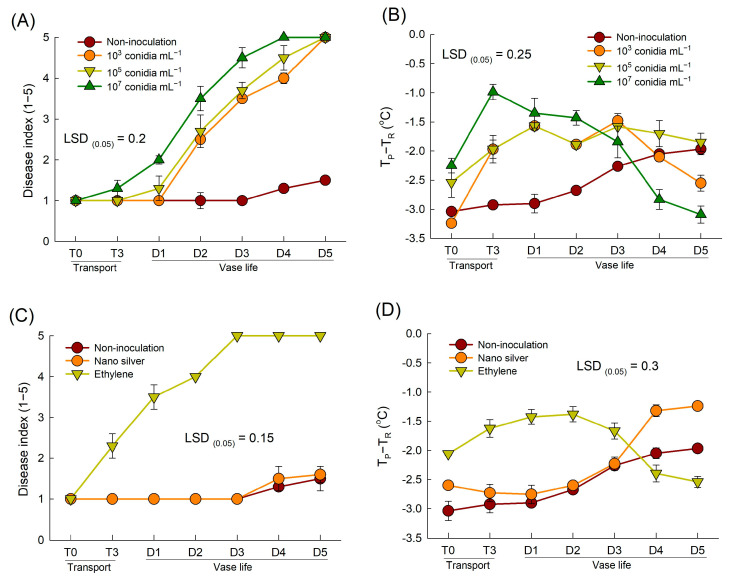
Effects of different inoculum concentrations of fungal conidia suspension (**A**,**B**) and chemical treatments (**C**,**D**) on disease index (**A**,**C**) and petal temperature difference (T_P_-T_R_) (**B**,**D**) of cut roses. T_P_ and T_R_ are the temperature of petals and reference plate. Cut flowers were sprayed with 30 mL of three concentrations of *Botrytis cinerea* suspension (10^3^, 10^5^, and 10^7^ conidia mL^−1^) to induce gray mold disease. Non-inoculated flowers were sprayed with 30 mL of distilled water. For chemical treatments, cut roses were treated with ethylene or nano silver before inoculating with *Botrytis cinerea*. Disease index was classified into five levels as 1, none; 2, slight symptoms (≤3%); 3, moderate symptoms (3–10%); 4, severe symptoms (11–50%); and 5, death of flowers (>50%). Disease index and petal temperature were evaluated on days 0 and 3 of transport (T0 and T3) and days 1–5 (D1–D5) of vase period. Data are shown as means ± SE (*n* = 24 for (**A**,**C**), and 12 for (**B**,**D**)).

The concentration of the *B. cinerea* suspension affected the severity of disease symptom and thermographic responses of rose petals significantly (Figure 4A,B). The DI in cut roses increased as the inoculum concentration in the conidial suspension increased (Figure 4A). The petal temperatures of all inoculated flowers transportation (T3) and days 1–3 of the vase period) (Figure 4B). Inoculation with the highest concentration of fungal conidia (10^7^ conidia mL^−1^) resulted in the highest petal temperature at the early stages of *B. cinerea* development (Figure 4B). However, at the late stage (days 4 and 5 of the vase period) of disease infection, the highest inoculum concentration caused decay and subsequent wetting of the petals; thus, the petal temperature was lower than those at other concentrations (Figure 4B).

Previous studies have shown that ethylene exposure increases *B. cinerea* susceptibility, while nano silver inhibits the development of *B. cinerea* in cut flowers [6,12]. Similarly, in the present study, ethylene exposure induced GMD in cut roses, whereas nano silver-treated flowers showed only slight disease symptoms during the late stage of the vase period (Figure 4C). These differences led to variation in petal temperature following *B. cinerea* inoculation. The petal temperatures of ethylene-treated flowers remained consistently 1.3–2.0 °C higher than those of non-inoculated flowers until day 3 of the vase period, after which the temperatures tended to decrease (Figure 4D). In contrast, the change in the petal temperature of nano silver-treated flowers resembled that of the non-inoculated flowers (Figure 4D). Overall, these results suggested that *B. cinerea* infection increased the petal temperature of cut roses during the early stages of pathogenesis.

### 2.4. Associations of the Petal Temperature with Fungal Biomass and Disease Index

**Figure 5:**
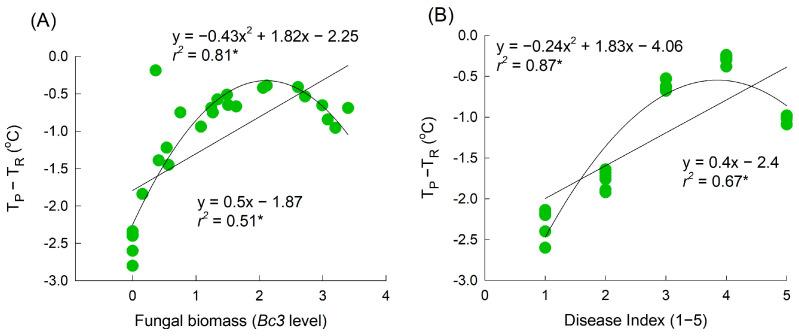
Regression between the petal temperature difference (T_P_-T_R_) and fungal biomass on petals (**A**) and disease index (**B**). T_P_ and T_R_ are the temperature of petals and reference plate. Fungal biomass was evaluated by *Bc3* level amplified by qRT-PCR from *Botrytis cinerea* gDNA. GMD index was classified into five levels as 1, none; 2, slight symptoms (≤3%); 3, moderate symptoms (3–10%); 4, severe symptoms (11–50%); and 5, death of flowers (>50%). Asterisk (*) represents a significant difference at *p* = 0.05.

The results of the above experiments demonstrated that the average petal temperature increases consistently over the course of disease infection. Linear regression analyses revealed that the change in petal temperature in *B. cinerea*-infected flowers increased proportionally with the DI and fungal biomass (Figure 5A,B). However, higher values of fungal biomass and DI tended to be associated with a lower petal temperature (Figure 5A,B). A quadratic regression analysis indicated that petal temperature increased initially with the *B. cinerea* infection in petals but decreased after the cut roses were fully macerated by GMD (Figure 5A,B). Overall, a strong correlation was observed between GMD infection and changes in petal temperature. Consequently, the alteration in petal temperature serves as a vital factor in predicting the presence or absence of *B. cinerea* infection in cut rose flowers.

We detected a significant relationship between the disease incidence in petals and the temperature on day 3 of transport (Temp-T3) and day 1 of the vase period (Temp-D1) (*r*^2^ = 0.71; *p* < 0.01) (Table 1). Notably, Temp-D1 had a higher standard partial regression coefficient than that of Temp-T3 (Table 1), indicating that the incidence of GMD in the petals of cut roses primarily depended on the change in petal temperature one day before the appearance of visible symptoms. Additionally, there was a significant negative correlation between Temp-D1 and VL of cut rose flowers (Supplementary Figure S2). 

## 3. Discussion

Postharvest cut roses are susceptible to various postharvest pathogens, and one common issue is GMD caused by the fungal pathogen *B. cinerea* [6,7,8]. Infection by *B. cinerea* can induce the ethylene production, lead to physiological disorders, and block water-conducting vessels in flower stems [2,3,4,5,6,7,8]. These factors make cut roses vulnerable to wilting and other physiological problems, ultimately shortening the VL of flowers. In this study, *B. cinerea* infection increased flower petal temperature and disturbed the delicate water balance in cut roses, which is essential for maintaining freshness, promoting flower opening, and extending the VL. *B. cinerea* inoculation also decreased the flower diameter and reduced the VL of cut flowers by 3.1 d compared to those of non-inoculated flowers. Additionally, GMD growth induces ethylene and ROS production by up-regulating the expression of genes related to ethylene biosynthesis and ROS production in rose petals. Therefore, prediction and early detection of GMD in cut roses are crucial for reducing postharvest losses and guaranteeing the longevity and marketability of rose flowers.

Thermal imaging is a non-contact method that converts the radiation emitted by an object captured by an infrared camera into surface temperature [16]. Recently, thermal imaging has been used to monitor temperature changes on plant surfaces following disease infections [23,24,25,26,27,29]. In this study, we used thermography to detect changes in the petal temperature of cut roses after inoculation with the necrotrophic pathogen, *B. cinerea*. Flower petals in most species are devoid of stomata [14]. Stomata, instead, are present on the leaves and stem [14]. Therefore, tissue temperature is independent of stomatal regulation in petals, but not in leaves or stems. Thus, indeed, the ideal tissue to study temperature differences is petals, where tissue temperature is independent of stomatal opening state. In tissues, where stomata are present, stomatal opening state (thus transpiration) dominates its temperature. Interestingly, thermal imaging revealed *B. cinerea* infection in rose petals one day earlier than visual observation. Variation in the petal temperature observed during the vase period was closely correlated with the development of *B. cinerea* in the petals. Specifically, we observed an initial increase in petal temperature after fungal infection, followed by a decrease in the late stages of GMD in the inoculated flowers. These results revealed that *B. cinerea* infection altered the petal temperature significantly, and the thermal responses of the petals changed dynamically over time. Fungal diseases have been detected before visible symptoms appear in grapevines, cucumbers, and potted rose plants using thermography [23,24,26,27,31]. However, the thermal responses of plant tissues vary during pathogenesis. For instance, infection with *Pseudoperonospora cubensis* in cucumber leaves initially results in a decreased leaf temperature due to water-soaked spots; however, as necrotic lesions form, the leaf temperature increases [27]. Conversely, infection with *Peronospora sparsa* in rose plants significantly increases the leaf temperature during pathogenesis, although this increase is lower when necrotic lesions are observed [23,24]. In this study, the initial increase in petal temperature during the early stages of GMD might be the result of host defense responses related to the activation of ethylene synthesis and ROS [32,33]. *B. cinerea* infection induced the expression of ethylene biosynthesis- and ROS-related genes in rose petals, which may generate heat as a byproduct of the plant defense, resulting in an increased petal temperature. Additionally, the mRNA levels of the aquaporin gene *RhTIP1* decreased rapidly when rose petals displayed necrotic lesions. This decrease may disrupt water transport and reduce the water content in petal tissues over a broader area, contributing to increased petal temperature [26,27]. However, at the late stage of the infection, *B. cinerea* leads to petal tissue rotting and a drop in petal temperature [25]. The severity of *B. cinerea* conidia germination and petal tissue rotting increased as the conidial concentration increased, resulting in an early temperature increase in the first stages and a rapid temperature decrease in the late stages at higher concentrations. These results align with those of a previous study demonstrating that inoculation with higher concentrations of *Venturia inaequalis* led to a sharp decrease in the apple leaf surface temperature, owing to rapid and intense damage to leaf tissues at the late stages of pathogenesis [25].

Ethylene induces rapid germination and development of *B. cinerea* in petals, resulting in a dramatic increase in petal temperature during the first stages and a rapid reduction in surface temperature during the late stages of the disease. In contrast, nano silver delayed the growth of *B. cinerea* in cut rose flowers, resulting in lower petal temperatures than those in ethylene-treated flowers. In the present study, the DI showed a slight increase in non-inoculated and nano silver-treated flowers during the late stage of the vase period. Mild disease symptoms in the petals of non-inoculated and nano silver-treated flowers appeared after the emergence of other senescence symptoms, such as wilting or bent neck. Therefore, the increase in petal temperature in non-inoculated and nano silver-treated flowers during the late stage of the vase period may be attributed to natural senescence, rather than to the development of *B. cinerea*.

Strong correlations between the petal area damaged by GMD, fungal biomass, and petal temperature were found in this study. However, a quadratic analysis provided a better description of the regression curve, particularly as the disease progressed to the macerated stage (where the infected area exceeded 50% or the *Bc3* level exceeded 2.5). This stage involves severe damage or the death of petal tissues, which is associated with petal senescence and the presence of wet petal tissues. These physiological changes ultimately lead to a reduction in petal tissue vitality and a decrease in petal temperature. A similar pattern has been observed in cucumber leaves infected with downy mildew [25,26,27,31]. In the present study, the petal temperature increased in the infected flowers on day 3 of transport and day 1 of the vase period (i.e., two and one days before early visible symptoms appeared, respectively). This makes petal temperature a suitable factor for distinguishing healthy flowers from infected flowers. The results of the multiple regression analysis also indicated that the incidence of diseased petals was primarily dependent on the petal temperature on day 1 of the vase period. Additionally, the VL of cut rose flowers showed a strong negative correlation with petal temperature on day 1 (Supplementary Figure S2). These results demonstrate that thermography can be used to detect *B. cinerea* infection in rose petals before disease symptoms become visible to the naked eye. Early detection using thermal imaging could be a valuable technique for implementing timely GMD management strategies and mitigating the spread of *B. cinerea* and its impact on cut rose flowers.

Although the thermographic technique proved to be a powerful tool for GMD detection in cut roses in this study, it had a limited in capacity for diagnosis. An increase in the temperature of the petals of cut roses may result from normal physical damages during the vase period. Additionally, certain pathogens can temporarily reduce the plant surface temperature [28]. Under stressful conditions, thermal imaging data can lead to inaccurate disease detection and evaluations of disease incidence and symptom severity, or even misdiagnosis. Furthermore, the difference in temperatures between diseased and healthy tissues is influenced by environmental conditions during measurement [26]. Therefore, combining thermography with other non-destructive techniques that provide additional spectral information, such as multispectral or hyperspectral imaging, is necessary for accurate disease detection and identification. Recent studies showed that the leaf water status estimation in *Spathiphyllum wallisii* and chrysanthemum is based on the visible portion of the electromagnetic spectrum (400–700 nm) and multispectral data (400–1050 nm) in association with artificial neural networks and convolutional neural network predictive models [34,35]. Therefore, in further research, estimates of *B. cinerea* infection in petals in combination with estimates of hydration status would give a good estimation of the potential vase life of cut rose flowers.

In conclusion, the pre-symptomatic detection of GMD in cut rose flowers was successfully achieved using thermography. Petal temperature dynamics were associated with the development of the GMD. The incidence of GMD in the petals of cut roses primarily depended on the petal temperature on the first day of the vase period before the appearance of visible symptoms. Therefore, thermography is an effective technique for monitoring changes in petal temperature and serves as a practical method for the early detection of *B. cinerea* infection in cut roses.

## 4. Materials and Methods

### 4.1. Plant Materials

**Figure 6:**
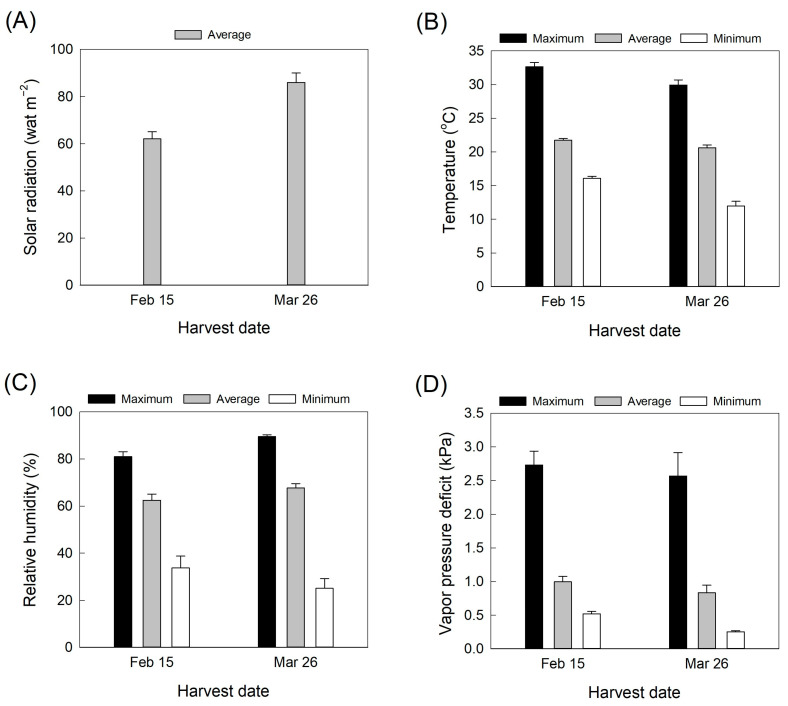
Changes in (**A**) solar radiation, (**B**) air temperature, (**C**), relative humidity, and (**D**) vapor pressure deficit in the greenhouse during cultivation. Environmental data are averages of daily values for 20 days before each harvest. Data are shown as mean ± SE (*n* = 20).

*Rosa hybrida* L. ‘Pink Beauty’ was obtained from a greenhouse in Jeonju, Korea. The rose plants were maintained in a greenhouse and were fertilized with a liquid nutrient solution (570 mL per rose plant per day) containing 0.97 mM Ca(NO_3_)_2_·4H_2_O, 2.5 mM NH_4_NO_3_, 0.09 mM KNO_3_, 1.502 mM MgSO_4_·7H_2_O, 0.67 mM KH_2_PO_4_, and small amounts of other minor elements such as B, Cl, Mo, Mn, Cu, Zn, and Fe. The air temperature, relative humidity, and light intensity in the greenhouse were recorded at 15 min intervals by using data loggers (WatchDog 1450, Spectrum Technologies, Aurora, IL, USA). The vapor pressure deficit in the greenhouse was calculated from the air temperature and relative humidity data. Solar irradiation was measured by a radiation sensor (SQ-100; Apogee Instruments, Inc., Logan, UT, USA) connected to the data loggers. Values used for analysis were averages of the daily values for the 20 days before each harvest (Figure 6A–D). Cut roses were harvested on 15 February and 26 March 2022. Symptomless rose flowers were collected and harvested at the commercial stage with outer petals bent out [3]. After harvest, cut flowers were placed in clean buckets containing tap water and transported to the laboratory within 3 h. The cut rose flowers were then held in distilled water and maintained in a controlled environment room at 23 ± 2 °C, a relative humidity of 50 ± 2%, and a photoperiod of 12 h with light supplied by fluorescence tubes at 20 µmol m^−1^ s^−1^ light density for vase life evaluation and further treatments. Distilled water was used, though less common from a practical stand point, since the tap water composition largely depends on the season, and the location [36].

### 4.2. Ethylene and Nano Silver Treatments

For ethylene treatment, cut roses were placed in distilled water and enclosed in a treatment chamber (462 L) at 23 ± 2 °C under dark conditions. Ethylene (46.2 mL of 10% ethylene) was injected into the treatment chamber to provide a final working concentration of 20 µL L^−1^ and cut roses were exposed to an ethylene atmosphere for 20 h. After exposure to ethylene, cut flowers were removed from the treatment chamber for fungal infection.

For nano silver treatment, cut roses were placed and sprayed with 20 mg L^−1^ nano silver (Shanghai HuZeng Nano Tech Co., Ltd., Shanghai, China) before fungal infection. The concentrations of ethylene and nano silver applied to the cut roses were based on our previous studies [6,12].

### 4.3. Fungal Suspension Preparation

*Botrytis cinerea* (KACC40573) was isolated from infected rose flowers at the Korean Agricultural Culture Collection, National Institute of Agricultural Sciences. For a pure culture, *B. cinerea* conidia were grown in potato dextrose agar medium (PDA, Difo Laboratories, Detroit, MI, USA) at 25 ± 1 °C for 14 days. The *B. cinerea* conidial suspension was obtained by adding 15 mL of distilled water to a culture Petri dish and gently sweeping the fungal colony surface with a sterile loop. Conidial clumps were removed from the suspension by gentle filtering through sterile gauze. Subsequently, the suspension concentration was estimated using a hemocytometer and diluted to 10^3^, 10^5^, and 10^7^ conidia mL^−1^ with distilled water for the inoculations.

### 4.4. Pathogen Inoculation and Transport Simulation

To evaluate the effects of different concentrations of the fungal suspension on disease infection and changes in temperature of the petals during the disease infection process, cut roses were held in distilled water and inoculated with three different concentrations of *B. cinerea* conidial suspension at 10^3^, 10^5^, and 10^7^ conidia mL^−1^. Each conidial suspension (30 mL) was sprayed onto cut flowers to induce GMD growth. Ethylene and nano silver-treated flowers were inoculated with 10^5^ conidia mL^−1^ suspension. Non-inoculated flowers were held and sprayed with 30 mL of distilled water.

After fungal inoculation, all cut flowers were stored for 3 days (T1 to T3) for simulating the transport conditions. The storage conditions have been described previously [6]. After the transport simulations, the flowers were recut using scissors to the same length (45 cm), and number of leaves (three upper leaves per stem). Each cut flower was then placed in a glass containing 400 mL of distilled water and held in a controlled environment room with the conditions set up following a previous study [6] for vase life and GMD progression evaluation and thermography analysis.

### 4.5. Disease Progression and Vase Life Evaluation

*Botrytis cinerea*-infected cut roses were evaluated daily for the development of GMD. The disease incidence rate in the petals was expressed as the proportion (%) of infected petals in cut flowers after inoculation. Disease severity was evaluated using the DI. The DI was assessed based on the percentage of petal area showing the necrotic lesion by GMD, as follows: 1, none; 2, slight symptoms (≤3%); 3, moderate symptoms (3–10%); 4, severe symptoms (11–50%); and 5, death of flowers (>50%).

To assess the influence of *B. cinerea* inoculation on the postharvest quality of cut roses, changes in fresh weight, water balance, and flower opening were measured daily. The measurements started daily at 10:30. The fresh weight and water uptake of cut roses were measured by weighing them during the vase period. The largest diameter of an individual flower and the diameter perpendicular to it were measured using digital calipers (CD-20APX, Mitutoyo Corporation, Kanagawa, Japan). The maximum flower diameter was calculated as a percentage of the initial flower diameter on day one of the vase period. Calculations of the water balance and maximum flower diameter of cut roses have been described previously [12]. The termination of VL in cut roses was determined when cut flowers exhibited moderate or severe disease symptoms on the petals [6,33]. In addition, the VL of cut flowers was terminated when petals or leaves wilted (≥50% of the leaf or petal turgor loss) and flower neck (peduncle) was bent [37,38].

### 4.6. Thermography Measurements, Data Collection, and Analysis

The thermal imaging of cut rose flowers was performed using the passive thermographic technique [38]. The infrared thermography equipment (T530) was placed in front of the cut roses. The distance between the cut roses and the infrared thermography camera lens was set to 45 cm for all treatments to minimize the influence of variation in distance. A reference plate (10 cm × 10 cm × 2 mm) with an infrared emissivity of 97% was installed at the same height and next to the cut flower. Thermal imaging of cut flowers in all treatments was taken in the same environmental conditions (23 ± 2 °C and 50 ± 2% of relative humidity). Thermal imaging of cut roses was performed on days 0 and 3 of transportation (T0 and T3), and on days 1, 2, 3, 4, and 5 of the vase period. Thermal images of cut flowers were exported to a computer using FLIR tools for petal temperature measurement. The temperature difference between the petal temperature (T_P_) and the temperature of the reference plate (T_R_) was determined as T_P_-T_R_ [38].

### 4.7. Fungal Biomass Evaluation and Gene Expression Analysis

Fungal biomass was assessed by the level of *Bc3* based on a previous study [6]. Briefly, fungal genomic DNA (gDNA) was isolated from the mycelia collected from *B. cinerea*-infected petals using the i-genomic BYF DNA Extraction Mini Kit (INTRON Biotechnology Inc., Gyeonggi-do, South Korea). *Bc3* was amplified from *B. cinerea* gDNA using quantitative real time PCR (qRT-PCR). *Bc3* levels were compared with those of *B. cinerea actin A*. The *actin A* and *Bc3* sequences as well as qRT-PCR conditions were described in a previous study [6].

Total RNA was isolated from 200 mg of rose petals using the GeneJET Plant RNA Purification Mini Kit (Thermo Fisher Scientific Baltics, Vilnius, Lithuania). cDNA was synthesized from 1 µg of total RNA using the XENO-cDNA Synthesis Kit (CELL TO BIO, Gyeonggi-do, South Korea) and a Bio-Rad PTC-100 Programmable Thermal Controller (MJ Research Inc., Hercules, CA, USA) as per the instruction manual. The transcript levels of ethylene biosynthesis-related genes (*RhACS2* and *RhACO1*), aquaporin-related genes (*RhTIP1*), and ROS-related genes (*RhCu/ZnSOD1* and *RhMnSOD1*) in petals of cut roses were analyzed using a BIO-RAD CFX Connect Real-Time System (Life Science, Hercules, CA, USA). *Rosa hybrida* actin 1 (*RhACT1*) was used as an internal control. The primer sequences used for qRT-PCR are listed in Table 2. The qRT-PCR reaction setting and conditions for gene expression analyses were described previously [34].

### 4.8. Experimental Design and Data Analysis

Twenty-four cut roses were used for each treatment. Experiments on the VL and DI were designed with 12 replicates (one flower per replicate). Thermal imaging measurements were performed with six replicates (one cut flower per replicate and cut flowers were part of the previously mentioned 12 replicates for VL and DI). The remaining twelve flowers were used for fungal biomass and gene expression analyses. The qRT-PCR analysis was conducted with three biological replicates. Data are presented as the mean ± standard errors (SE). All experiments were conducted twice in February and March 2022. Least significant difference tests (LSD; *p* = 0.05), analysis of variance, simple linear regression, quadratic regression, and multiple regression were used to analyze the data. Student’s *t*-test was performed for comparisons of means as well as standard analysis of variance at a significant level of 95%. Statistical analyses were performed using SPSS version 22.0 (IBM, Armonk, NY, USA).

## Figures and Tables

**Table 1:** Result of multiple regression analysis of the relationship of GMD incidence in petals and petal temperature.

Independent Variable	Partial Regression Coefficient	Standard Error	Standard Partial Regression Coefficient
Constant	76.78 **	7.94	
Temp-T3	10.48 *	4.17	0.28
Temp-D1	20.16 **	6.70	0.52
Temp-D2	6.01	5.77	0.17

Petal temperature was measured on day 3 (Temp-T3) and days 1 and 2 (Temp-D1 and Temp-D2) of the vase period. Asterisks (* and **) indicate significant differences at *p* < 0.05 and *p* < 0.01, respectively. Regression statistic: *r*^2^ = 0.7 ** (*n* = 72).

**Table 2:** Primer pairs used for amplification of cDNA by qRT-PCR analyses.

Gene (Accession Number)	Forward Primer	Reverse Primer	Size
*RhACS2* (AY803738.1)	5′-ATTTCTGGTTCCCGTTCCTT-3′	5′-TGGGCTTTCTCATAGGCATC-3′	147
*RhACO1* (AF441282.1)	5′-CGTTCTACAACCCAGGCAAT-3′	5′-TTGAGGCCTGCATAGAGCTT-3′	130
*RhCu/ZnSOD1* (KJ159891.1)	5′-TGCTGAGGTTTCTGTTGACG-3′	5′-GCAAGAGTTGGATGCGGTAT-3′	162
*RhMnSOD1* (KJ159890.1)	5′-GCAAGTTTAGTGCCCTTGCT-3′	5′-TGTACGAGAAAGAAAGCGCC-3′	147
*RhTIP1* (KF985188.1)	5′-TCTCTCCTACGTGGCATCCT-3′	5′-GACCACCTCTGCTTTTGCTC-3′	109
*RhACT1* (KC514918.1)	5′-GTTCCCAGGAATCGCTGATA-3′	5′-ATCCTCCGATCCAAACACTG-3′	116

## Data Availability

Data are contained within the article.

## Supplementary Materials

The following supporting information can be downloaded at: https://www.mdpi.com/article/10.3390/plants12244087/s1, Figure S1: Effects of *Botrytis cinerea* infection on changes in the relative expression of *RhCu/ZnSOD1* in cut rose flowers. Gene expression levels in cut roses were analyzed at day 0 of transport (T0) and days 1 (D1), 2 (D3), and 5 (D5) of the vase period. Cut flowers were sprayed with 30 mL of *B. cinerea* suspension (10^5^ conidia mL^−1^) to induce gray mold disease. Non-inoculated flowers were sprayed with 30 mL of distilled water. Data are shown as means ± SE (*n* = 6); Figure S2: Correlation coefficient (*r*) values for the relationships of petal temperature and vase life of cut roses. Data were subjected to Pearson correlation analysis (*n* = 72). Petal temperature was measured at days 0 (Temp-T0) and 3 (Temp-T3) of transport and days 1 (Temp-D1), 2 (Temp-D2), 3 (Temp-D3), 4 (Temp-D4), and 5 (Temp-D5) of the vase period. Asterisks (**) indicate a significant difference at *p* < 0.01.
