# Supplementary material for: Early Detection of Botrytis cinerea Infection in Cut Roses Using Thermal Imaging
Source: Plants (Basel). 2023 Dec 6;12(24):4087. doi: 10.3390/plants12244087 (PMC10748118; doi:10.3390/plants12244087)
Supplement: Supplementary file 1 [file plants-12-04087-s001.zip › plants-2743545-supplementary.pdf]

Supplementary Figures

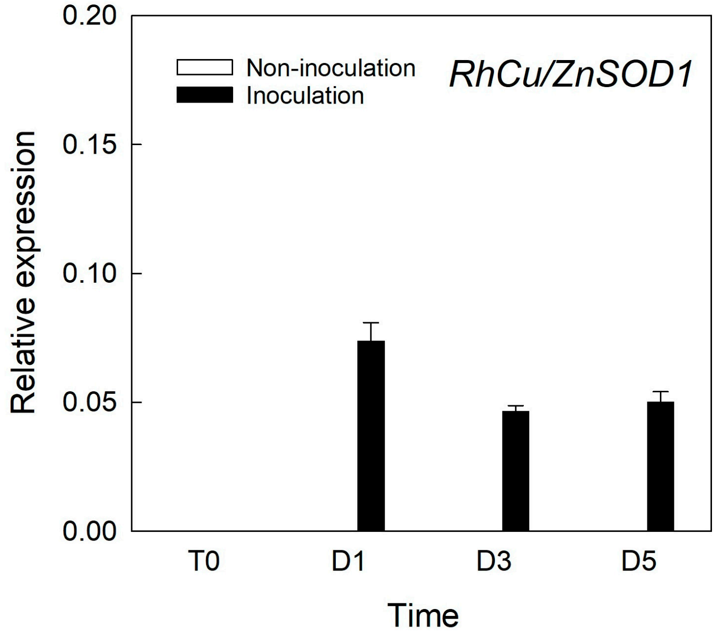

Figure S1. Effects of *Botrytis cinerea* infection on changes in the relative expression of *RhCu/ZnSOD1* in cut rose flowers. Gene expression levels in cut roses were analyzed at day 0 of transport (T0) and days 1 (D1), 2 (D3), and 5 (D5) of the vase period. Cut flowers were sprayed with 30 mL of *B. cinerea* suspension (10^5 conidia mL^{-1}) to induce gray mold disease. Non-inoculated flowers were sprayed with 30 mL of distilled water. Data are shown as means ± SE (n = 6).

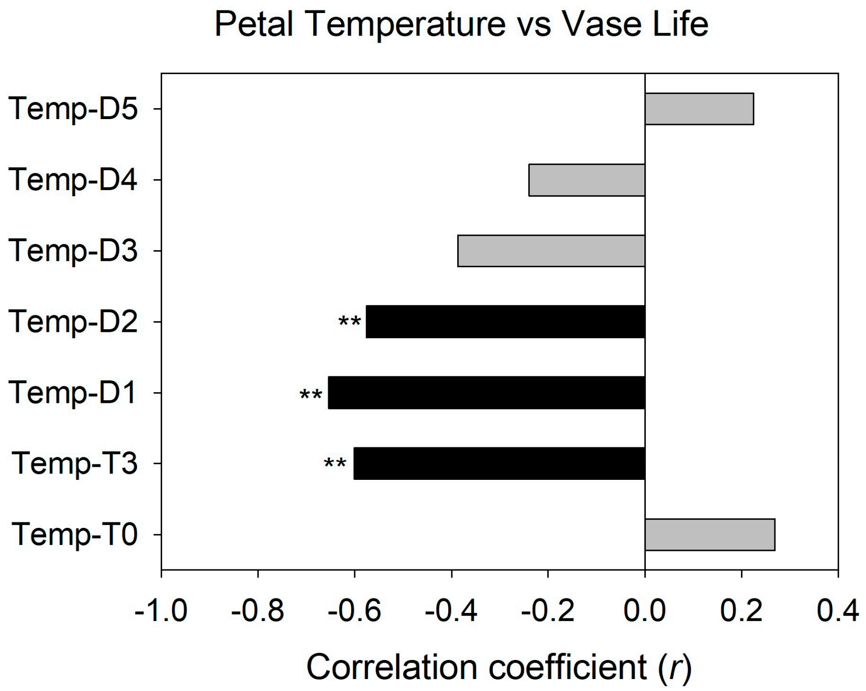

Figure S2. Correlation coefficient (*r*) values for the relationships of petal temperature and vase life of cut roses. Data were subjected to Pearson correlation analysis ($n = 72$). Petal temperature was measured at days 0 (Temp-T0) and 3 (Temp-T3) of transport and days 1 (Temp-D1), 2 (Temp-D2), 3 (Temp-D3), 4 (Temp-D4), and 5 (Temp-D5) of the vase period. Asterisks (**) indicate a significant difference at $p < 0.01$.
